# Genomic Sequence and Characteristics of EmiRose, a Bacteriophage Isolated on Corynebacterium flavescens

**DOI:** 10.1128/mra.00080-22

**Published:** 2022-03-02

**Authors:** Emily Rose Davis, Blesyn Perry-Richardson, Aleem Mohamed, Ilzat Ali, Danielle Heller, Viknesh Sivanathan

**Affiliations:** a Department of Biological Sciences, University of Maryland Baltimore County, Baltimore, Maryland, USA; b Public Safety Program, University of Maryland Global Campus, Adelphi, Maryland, USA; c Department of Science Education, Howard Hughes Medical Institute, Chevy Chase, Maryland, USA; DOE Joint Genome Institute

## Abstract

Bacteriophage EmiRose is a siphovirus infecting Corynebacterium flavescens. The EmiRose genome is 37,431 bp long and composed of 47 protein-coding genes. Based on gene content similarity, EmiRose is not closely related to any previously sequenced bacteriophages in the actinobacteriophage database to date, including other corynebacteriophages. EmiRose is classified as a singleton.

## ANNOUNCEMENT

**FIG 1:**
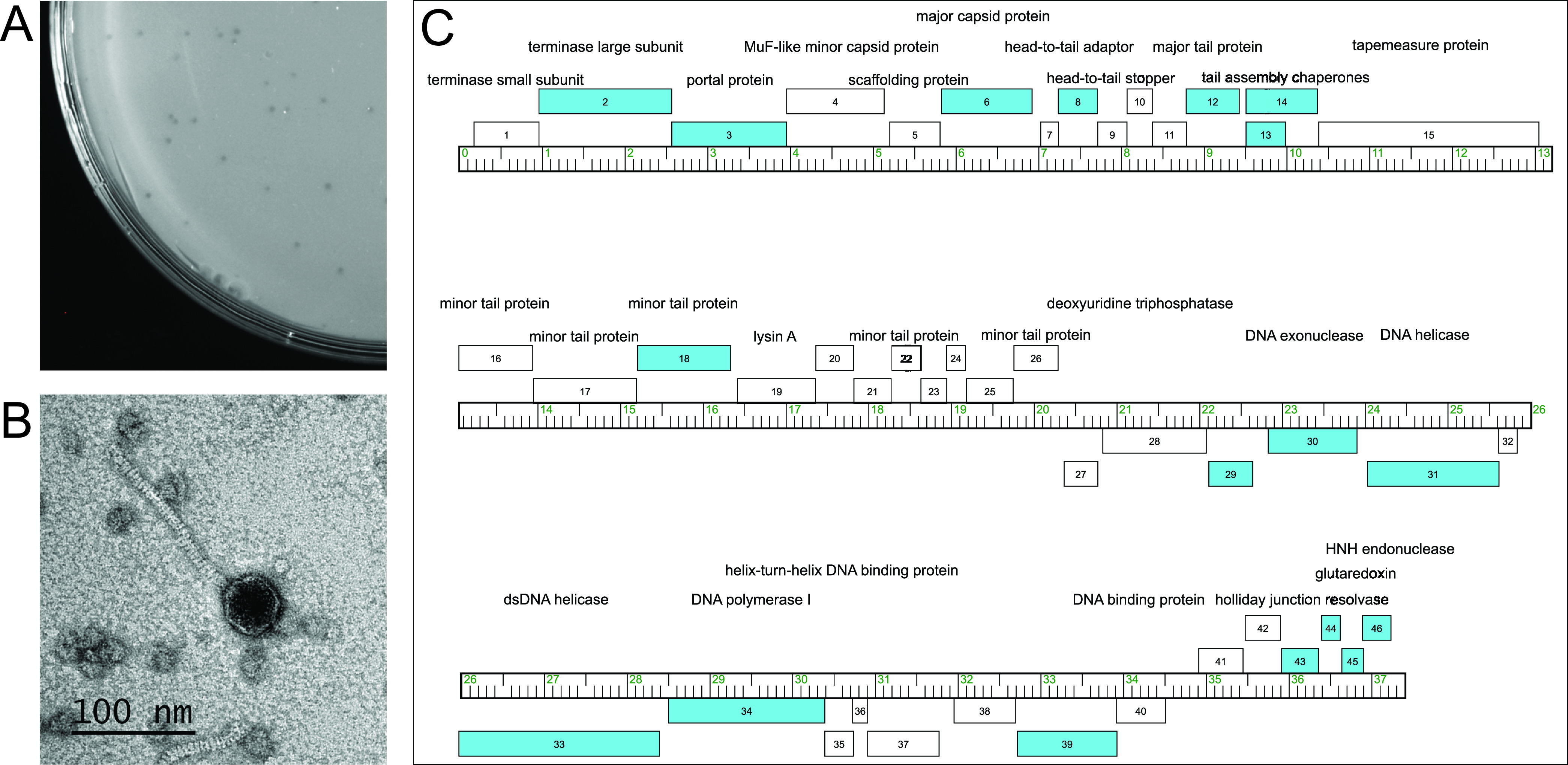
Characteristics of corynebacteriophage EmiRose. (A) EmiRose forms small and turbid plaques 1 to 2 nm in diameter after 48 h of incubation at 30°C. (B) A negative-stain transmission electron micrograph showing the *Siphoviridae* morphology of EmiRose, with a flexible tail 141 to 144 nm long attached to an isometric capsid 43 to 44 nm in diameter, based on measurement of 2 particles. (C) A map of the EmiRose genome, with genes presented as boxes along a genome ruler (in kilobases). The boxes above and below the ruler represent genes that are transcribed rightward and leftward, respectively. The blue and white boxes represent genes coding for proteins with and without homologs shared with other bacteriophages, respectively. The gene number is presented within each box.

Bacteriophages play complex roles in bacterial pathogenesis. In the case of Corynebacterium diphtheriae, severe illness is caused by bacterial expression of a prophage-encoded toxin (1). Conversely, bacteriophages are increasingly being considered for use as therapeutics for treating infections caused by diverse bacterial pathogens (2). Given this multifaceted role in medicine, the isolation and genetic characterization of new bacteriophages is important. Here, we report on EmiRose, a corynebacteriophage isolated from topsoil collected from a sidewalk in West Hollywood, CA (global positioning system [GPS] coordinates, 34.076042 N, 118.3639 W), using standard methods (3). Briefly, EmiRose was extracted by washing the soil sample with peptone-yeast extract-calcium (PYCa) liquid medium supplemented with 0.1% dextrose. EmiRose was then enriched in the filtered (0.2-μm pore size) wash and purified with three successive rounds of plating on Corynebacterium flavescens strain ATCC 10340 at 30°C. Top agar overlay of EmiRose resulted in small turbid plaques 1 to 2 mm in diameter after 48 h of incubation at 30°C (Fig. 1A). Negative-stain transmission electron microscopy revealed EmiRose to be a siphovirus with a flexible tail 141 nm long and an isometric capsid 43 nm in diameter (Fig. 1B).

Genomic DNA was isolated from EmiRose using the Promega Wizard DNA cleanup kit, prepared for sequencing using the NEBNext Ultra II FS kit, and sequenced using an Illumina MiSeq instrument, yielding 1,025,624 single-end 150-bp reads constituting 4,110-fold coverage of the genome. The untrimmed reads were assembled as a single contig using Newbler v2.9 and checked for completeness and genomic termini using Consed v29 as previously described (4), yielding a genome 37,431 bp long with 9-nucleotide 3′ single-stranded cohesive ends (5′-CCCTCCGGT-3′) and an average G+C content of 56.4%. Based on its gene content similarity (GCS) of 35% or higher to sequenced bacteriophages present in the actinobacteriophage database, PhagesDB (5), using the PhagesDB GCS tool and previously described criteria (6), EmiRose is not closely related to any bacteriophage, including 19 other corynebacteriophages isolated on Corynebacterium xerosis and Corynebacterium vitaeruminis. EmiRose is thereby classified as a singleton. The genome was annotated using DNA Master (http://cobamide2.bio.pitt.edu/), Glimmer v3.02 (7), GeneMark v3.25 (8), BLAST (9), HHpred (10), Aragorn v1.1 (11), and tRNAscan-SE v2.0 (12), all with default parameters. The resulting annotation process revealed a total of 47 protein-coding genes, for which 26 proteins could be assigned putative functions (Fig. 1C). Genes coding for functions in DNA packaging (1, 2), virion structure and assembly (3 to 6, 8, 9, 12 to 18, 22, 26), and lysis (18) occupy the left half of the genome and are transcribed rightward. DNA metabolism genes are dispersed throughout the right half of the genome, where the genes are transcribed leftward, although the six rightmost genes are transcribed rightward. EmiRose does not encode identifiable integrase or immunity repressor functions, and it is unlikely to establish lysogeny. Similarly, no identifiable toxin is encoded by EmiRose, and we note that gp4, encoding a MuF-like protein that is toxin associated in some other bacteriophages, lacks the toxin domain. Based on the protein sequence similarity, as previously described (13), only 18 EmiRose proteins have homologs shared with other bacteriophages (https://phagesdb.org/). These include 17 proteins with homologs found broadly among actinobacteriophages and 1 (minor tail protein, gp17) shared only with the singleton bacteriophage GMA4, which was isolated on Gordonia malaquae.

### Data availability.

EmiRose is available at GenBank under accession no. MN586033 and Sequence Read Archive (SRA) accession no. SRX5653831.
